# Validating the representation of distance between infarct diseases using word embedding

**DOI:** 10.1186/s12911-022-02061-8

**Published:** 2022-12-07

**Authors:** Daiki Yokokawa, Kazutaka Noda, Yasutaka Yanagita, Takanori Uehara, Yoshiyuki Ohira, Kiyoshi Shikino, Tomoko Tsukamoto, Masatomi Ikusaka

**Affiliations:** 1 Department of General Medicine, Chiba University Hospital, 1-8-1 Inohana, Chuo-Ku, Chiba City, Chiba 260-8677 Japan; 2 Department of General Internal Medicine, St. Marianna University School of Medicine, 2-16-1 Sugao, Miyamae-Ku, Kawasaki City, Kanagawa Japan

**Keywords:** Infarct disease, Word2Vec, Pivot and cluster strategy, Clustering, ICD-10

## Abstract

**Background:**

The pivot and cluster strategy (PCS) is a diagnostic reasoning strategy that automatically elicits disease clusters similar to a differential diagnosis in a batch. Although physicians know empirically which disease clusters are similar, there has been no quantitative evaluation. This study aimed to determine whether inter-disease distances between word embedding vectors using the PCS are a valid quantitative representation of similar disease groups in a limited domain.

**Methods:**

Abstracts were extracted from the Ichushi Web database and subjected to morphological analysis and training using Word2Vec, FastText, and GloVe. Consequently, word embedding vectors were obtained. For words including “infarction,” we calculated the cophenetic correlation coefficient (CCC) as an internal validity measure and the adjusted rand index (ARI), normalized mutual information (NMI), and adjusted mutual information (AMI) with ICD-10 codes as the external validity measures. This was performed for each combination of metric and hierarchical clustering method.

**Results:**

Seventy-one words included “infarction,” of which 38 diseases matched the ICD-10 standard with the appearance of 21 unique ICD-10 codes. When using Word2Vec, the CCC was most significant at 0.8690 (metric and method: euclidean and centroid), whereas the AMI was maximal at 0.4109 (metric and method: cosine and correlation, and average and weighted). The NMI and ARI were maximal at 0.8463 and 0.3593, respectively (metric and method: cosine and complete). FastText and GloVe generally resulted in the same trend as Word2Vec, and the metric and method that maximized CCC differed from the ones that maximized the external validity measures.

**Conclusions:**

The metric and method that maximized the internal validity measure differed from those that maximized the external validity measures; both produced different results. The cosine distance should be used when considering ICD-10, and the Euclidean distance when considering the frequency of word occurrence. The distributed representation, when trained by Word2Vec on the “infarction” domain from a Japanese academic corpus, provides an objective inter-disease distance used in PCS.

## Background

A common clinical reasoning strategy is to recall a disease and check whether the patient history obtained is consistent with the disease [1]. Physicians take the first step, i.e., disease recall, in terms of prior probabilities that vary according to age and sex and the function and location of the medical facility. Based on these prior probabilities, they often recall one or two diagnoses and consider them based on a patient’s symptoms and characteristic findings in clinical practice. Sometimes, a list of differential diagnoses is required as competing hypotheses for a given diagnosis. Differential diagnosis lists are generated by specific rules, such as diseases with other pathologies occurring in the same organ, diseases occurring in anatomically adjacent organs, and diseases with similar pathologies but occurring in multiple organs. However, when physicians generate disease recall and differential diagnosis lists, there is always a chance for diagnostic errors due to heuristic bias.

The pivot and cluster strategy (PCS) can be used to avoid such bias [2]. In PCS, clinicians simultaneously recall a differential diagnosis list that approximates one of the recalled diagnoses based on intuition. The process involved in PCS is as follows [2, 3]: First, a clinician designates the initial diagnosis (pivot) as the most probable hypothesis through an intuitive or analytical process based on history and physical examination as well as their knowledge and experience. Second, the clinician forms a disease cluster around the pivot to obtain a collection of differential diagnoses. Any disease can be a pivot. The list of differential diagnoses can be shortened or expanded according to the overlaps and differences between pivots and their clusters.

Pivot designation is intuitive and can lead to diagnostic errors due to heuristic cognitive bias. Pivot designation is influenced by the frequency of disease, which depends on the function and location of the medical institution, and the physician’s specialty and case experience [4]. However, Shimizu et al. argued that in PCS, the automatic and simultaneous recall of clusters close to the pivot’s clinical presentation removes bias and improves diagnostic accuracy by preventing early closure [2]. They stated that PCS is also useful when teaching novice students. In PCS, the teacher, an experienced clinician, assigns a virtual distance from the pivot to the differential diagnosis and translates the list of differential diagnoses into a two-dimensional visual representation. This helps the learner represent the degree of concurrence with the patient’s clinical symptoms.

Shimizu et al. stated that, in PCS, pre-prepared clusters can automatically and quickly be recalled in batches. Nevertheless, a question arises: “what do they mean by ‘pre-prepared clusters’ in actual clinical practice?” We contend that pre-prepared clusters may be based on intuition stemming from the accumulated experience of each clinician or on textbook knowledge. In general, disease clusters based on similar pathophysiologies are likely to have similar medical characteristics. Despite physicians being empirically aware of “similarities” among disease groups, there has been no quantitative presentation of these disease groups. Exhaustive clusters are required because user-friendly, limited clusters based on physicians’ experience can be a source of cognitive bias. However, it is difficult to prepare uniform clusters for all pivots, and even if one could, it would be impractical to use them for a physician’s reasoning. Conversely, the differential diagnosis generator is a computer-generated list of differential diagnoses, which reportedly allows clinicians to reconsider their diagnoses [5]. If we can quantitatively represent clusters, the accuracy of the differential diagnosis generator is expected to improve significantly.

We collected documents related to “infarction” from the corpus of Ichushi Web, a database of medical articles, used Word2Vec to learn word associations from the collected articles, and found that the pathophysiological and anatomical features of “infarction” are retained in the distributed representation [6]. In our previous article (under peer review), we suggested that “brain infarction” and “myocardial infarction” have different vectors and that some vectors share greater similarity if they are diseases of the same organ. Word2Vec is an unsupervised learning system that uses neural networks and a tool to compute distributed representations of words [7]. Word2Vec is effective in capturing semantic relatedness and similarity relations among medical terms [8]. FastText is a library created by Facebook's AI Research Laboratory for learning word embeddings and exploiting subword information to construct word embeddings [9]. GloVe is an unsupervised learning algorithm with aggregated global word–word co-occurrence statistics for obtaining vector representations of words [10].

The present study examined the validity of the similarity (inter-diseases distance) calculated using our learned word embedding vectors as candidates for clusters in PCS. To evaluate the validity, we verified the agreement rate with the International Statistical Classification of Diseases and Related Health Problems (ICD), an external classification list provided by the World Health Organization. The 10th edition (ICD-10) is available in Japanese. It contains codes for diseases, their signs, and symptoms. Its hierarchical structure is divided into 22 chapters, and the circulatory system diseases (I00-99) include many strokes and myocardial infarctions. It is highly reliable because it is based on human judgment informed by pathology and anatomical relationships.

Is clustering by word embedding vectors similar to ICD-10 clustering? If demonstrated, this hypothesis would provide reasonable grounds for a quantitative and objective presentation of clusters by word embedding vectors in medical corpora.

## Objectives

This study aimed to determine whether the inter-disease distances between word embedding vectors using the PCS are a valid quantitative representation of similar disease groups in a limited domain. First, we attempted to obtain a distributed representation of words using Japanese medical journal abstracts and Word2Vec. Next, we examined the validity of the representation in terms of internal and external validity corresponding to ICD-10 in the clustering of word vectors.

## Methods

### Subjects

We extracted abstract texts from the medical journal database of the NPO Japan Medical Abstracts Society, Ichushi (requires registration) by searching for the word “infarction,” and we set the search criteria to show case report articles that contain an abstract [6]. We limited our search to the infarction domain because it involves the same pathological changes in multiple organs. Many researchers have used abstracts from Ichushi Web as subjects for their literature review [11–13]. We inserted spaces between words and performed morphological analysis on the abstract texts to produce word sequences converted into a standard form.

In Japanese, unlike English, there are no spaces between words. Therefore, to separate words, it is necessary to insert a space while checking against a dictionary. MeCab [14] was used for inserting spaces and for morphological analysis, and mecab-ipadic-NEologd [15] and ComeJisyo [16] were used as the dictionaries. ComeJisyo is a dictionary for MeCab that leaves a space between the words that represent terms used in medical facilities. After morphological analysis, we exclusively extracted terms consisting of nouns, adjectives, adverbs, and verbs. Numeric expressions are typically not crucial in Japanese natural language processing. We excluded a number of nouns from the sequences, e.g., numerals indicating a subject’s height and weight, and the dosage of medication.

### Model learning

We trained the word sequences using the Gensim Word2Vec package [17]. Word2Vec is available in two different forms: skip-gram and continuous bag of words (CBOW). In previous reports, Word2Vec, specifically the skip-gram architecture, achieved the highest score on three of four rated tasks: analogy-based operations, odd one similarity, and human validation [18]. Skip-grams also performed better in biomedical studies [19–22].

The skip-gram model is a simple neural network with one hidden layer and an output layer with a softmax activation function. This model can accurately predict the words before and after an input word. Given some text, a target word is selected within a window. The training data for the neural network consists of pairwise combinations of the target word and all the other words in the window. The output layer yields a vector of the same size as the input, and each element in the vector consists of a probability indicating the similarity between the target word and other words in the vocabulary.

FastText is also a skip-gram model based on subwords that can handle out-of-vocabulary words. GloVe is a method that learns by combining word embeddings from the local window intra-word context with a document-wide global co-occurrence matrix. To confirm the differences between the learning models, we trained FastText and GloVe on the same corpus. The word embedding vectors learned by each algorithm were analyzed using the Gensim package.

This study used a skip-gram algorithm based on previous Word2Vec and FastText studies, with a vector of 200 dimensions. We set the window size as 5, minimum count as 5, and number of iterations as 100. Words containing “infarction” were extracted from the vocabulary used for training. We retained only the words found in ICD-10 that matched perfectly and used the set of words as the standard name list.

### Clustering and evaluating with internal validity measures

Each word in the standard name list had a vector of 200 dimensions. We used the hierarchical clustering in ICD-10 based on each vector component. As the figures in later sections show, (the terms in parentheses follow a lower-case naming convention) we used distance definitions (metric), including Euclidean (euclidean), city-block (cityblock), standard Euclidean (seuclidean), cosine, correlation, Chebyshev (chebyshev), Canberra (canberra), and Brady Curtis (braycurtis) distances. We used update methods (method), including single linkage (single), complete linkage (complete), group average (average), weighted average (weighted), median, Ward’s (ward), and centroid. With this combination of metrics and methods, we first calculated the cophenetic correlation coefficient (CCC), which is an internal validity measure of clustering. We then created a dendrogram using the combination with the highest CCC value when using Word2Vec.

1 $$ cophenet\left( {Z, \,Y} \right) = \frac{{\mathop \sum \nolimits_{i < j} \left( {Y_{ij} - y} \right)\left( {Z_{ij} - z} \right)}}{{\sqrt {\mathop \sum \nolimits_{i < j} \left( {Y_{ij} - y} \right)^{2} \mathop \sum \nolimits_{i < j} \left( {Z_{ij} - z} \right)^{2} } }} $$

$$ Y:pairwise dis\tan ces,\, Z:dis\tan ces in the dendrogram, \,y and z:means of Y and Z, $$

$$ Y_{ij} and Z_{ij} : i - th and j - th components of Y and Z $$

### Evaluation with external validity scale

We evaluated the model with an external validity scale. External validity measures performance by matching the clustering structure to a priori information, i.e., true class labels [23, 24]. Each word in the standard name list was assigned its corresponding ICD-10 code. We counted the number of unique ICD-10 codes and calculated the adjusted rand index (ARI) [25, 26], normalized mutual information (NMI) [27], and adjusted mutual information (AMI) [28] as external validity measures when clustering the standard name list to that unique number. These are the most commonly used evaluation measures to assess the similarity between two sets [29]. The range of ARI is [− 1, 1], that of NMI is [0, 1], and that of AMI is [0, 1]; a larger value indicates a closer match between the two groups. AMI is appropriate when the clusters are of unequal sizes and contain smaller clusters than ARI [30]. We calculated the distances and external validity measures for each combination, and the update methods were the same as those mentioned in Sect. 2.3.

### Analytics

The analysis was performed on a terminal using Ubuntu 20·04·2 LTS, with an Intel Core i9-9960X CPU, 64 GB of primary memory, and two NVIDIA TITAN RTX GPUs with a total RAM of 48 GB that were linked using an NVLink bridge. The machine learning framework consisted of Python (3·6·9), Gensim (3·8·3), and scikit-learn (0·24 2), which is a module for machine learning in Python. We used FastText (0·9·2) and GloVe (1·2).

## Results

Since October 26, 2019, 15,513 abstracts have been extracted (Ichushi ID: 1,983,011,395 to 2,019,316,513). The insertion of spaces (as explained in Sect. 2.1) and morphological analysis led to 1,505,041 words and 46,602 unique words in the unlearned word sequences. The word sets containing “infarction” had 20,918 words, of which 71 were unique. The word sequences used for training word embedding vectors had 1,445,433 words, with 15,163 being unique. The word sets containing “infarction” and used for training word embedding vectors had 20,877 words, of which 54 were unique.

We can quickly implement PCS with distributed representation by running the “gensim.models.Word2Vec.similar_by_vector” method. This method can output any number of highly similar vectors to the input word embedding vector. The similarity is calculated using the cosine distance. Table 1 presents examples of clusters with myocardial infarction, cerebral infarction, liver cirrhosis, and dementia as Pivot. The corpus used in our study is based on the infarction disease domain, and the results are affected by co-occurrences within this domain. It is therefore difficult to include diseases distant from infarct diseases in the results.

**Table 1:** Examples of pivot and cluster strategy in word embeddings obtained using Word2Vec

Pivot	Myocardial infarction	Cerebral infarction	Liver cirrhosis	Dementia
Clusters (similarity)	Acute myocardial infarction (0.7064)	Lacunar infarction (0.5465)	Hepatocellular carcinoma (0.4837)	Alzheimer's dementia (0.4670)
	Acute inferior myocardial infarction (0.5480)	Cerebellar infarction (0.5333)	Hepatitis C (0.4303)	Parkinson’s syndrome (0.4639)
	Angina pectoris (0.5076)	Cerebral hemorrhage (0.4552)	Gastric cancer (0.3898)	Vascular dementia (0.4551)
	Ischemic heart disease (0.4883)	Transient ischemic attack (0.4155)	Esophageal varix (0.3750)	Depression (0.4056)

Table 2 summarizes the set of words containing “infarction” extracted exclusively from words that fully matched ICD-10 (the standard name list), the total number of words, and ICD-10 codes. The total number of words was 15,163, and there were 38 unique words. Twenty-one ICD-10 codes appeared; 16 of the 54 did not match an ICD-10 code. The words containing “infarction” that did not match ICD-10 were “infarction,” “hemorrhagic infarction,” “inferior infarction,” “post-myocardial infarction,” “postinfarction,” “inferior myocardial infarction,” “anterior infarction,” “anterior myocardial infarction,” “right ventricular infarction,” “infarctive,” “reinfarction,” “anterior septal myocardial infarction,” “pulmonary infarction disease,” “impending myocardial infarction,” “multi-infarct dementia,” “perioperative myocardial infarction,” “thrombotic infarction,” “impending infarction,” and “posterior myocardial infarction”.

**Table 2:** Standard name list: set of words containing “infarction” that precisely match ICD-10

Word	Number	ICD-10 code	Word	Number	ICD-10 code
Cerebral infarction	6834	I639	Pontine infarction	82	I635
Acute myocardial infarction	2301	I219	Medullary infarction	66	I635
Myocardial infarction	1754	I219	Acute anterior myocardial infarction	59	I210
Multiple cerebral infarction	524	I638	Asymptomatic cerebral infarction	43	I638
Pulmonary infarction	467	I269	Acute anterior septal myocardial infarction	31	I210
Old myocardial infarction	397	I252	Omental infarction	27	K550
Renal infarction	333	N280	Embolic infarction	22	I749
Lacunar infarction	327	I638	Placental infarction	20	O438
Splenic infarction	263	D735	Post-acute myocardial infarction ventricular septal perforation	19	I232
Cerebellar infarction	285	I635	Old inferior myocardial infarction	11	I252
Sequelae of cerebral infarction	255	I693	Perforating branch infarction	12	I635
spinal cord infarction	238	G951	Recurrent cerebral infarction	11	I639
Post-cerebral infarction	202	I693	Cerebral venous infarction	10	I636
Brainstem infarction	199	I635	Adrenal Infarction	9	E274
Old cerebral infarction	153	I693	Old anterior septal myocardial infarction	7	I252
Atherosclerotic cerebral infarction	120	I633	Acute lateral myocardial infarction	7	I212
Acute inferior myocardial infarction	119	I211	Cortical branch infarction	7	I635
Hepatic infarction	113	K763	Kidney infarction	7	N280
Hemorrhagic cerebral infarction	103	I638	Watershed infarction	6	I638
			Total: 38 words	15,163	21 codes

**Fig. 1:**
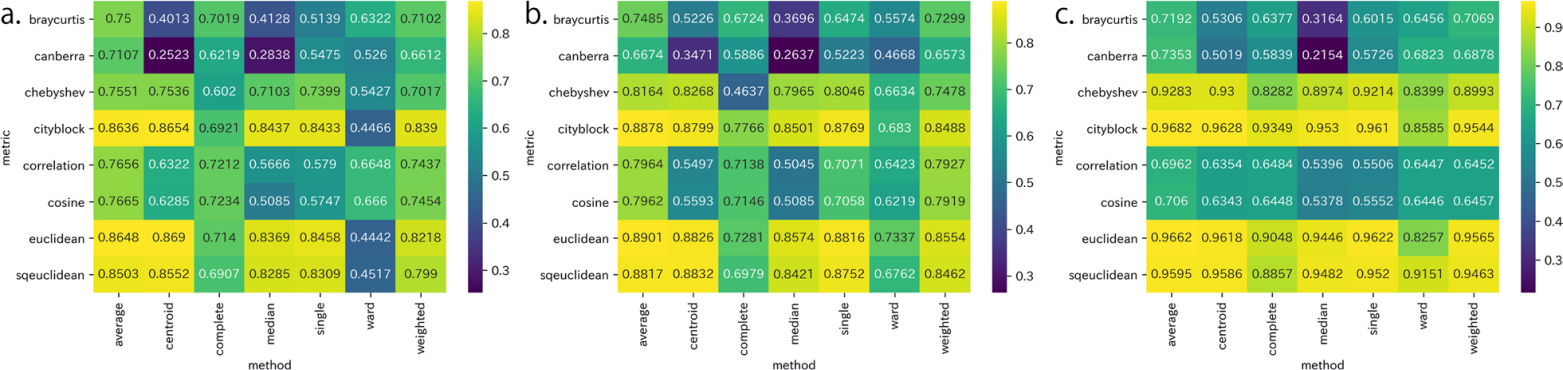
Internal validity measures with cophenetic correlation coefficients (CCCs) for the embedding vectors of **a** Word2Vec, **b** FastText, and **c** GloVe

Figure 1a shows the CCCs obtained using the embedding vectors of Word2Vec for each definition of the metric and update methods for the evaluation using the internal validity measure. When the metric was euclidean and the method was centroid, the CCC was maximal at 0.8690. Figure 1b and c show the CCCs obtained using the embedding vectors of FastText and GloVe, respectively. When the metric was euclidean and the method used was average, the CCC was maximal at 0.8901 using FastText. When the metric was cityblock and the method used was average, the CCC was maximal at 0.9682 using GloVe. Although the maximum metric and method differed, the generally high values for the euclidean, sqeuclidean, and cityblock metrics and the average and centroid methods were similar for all the learning algorithms.

**Fig. 2:**
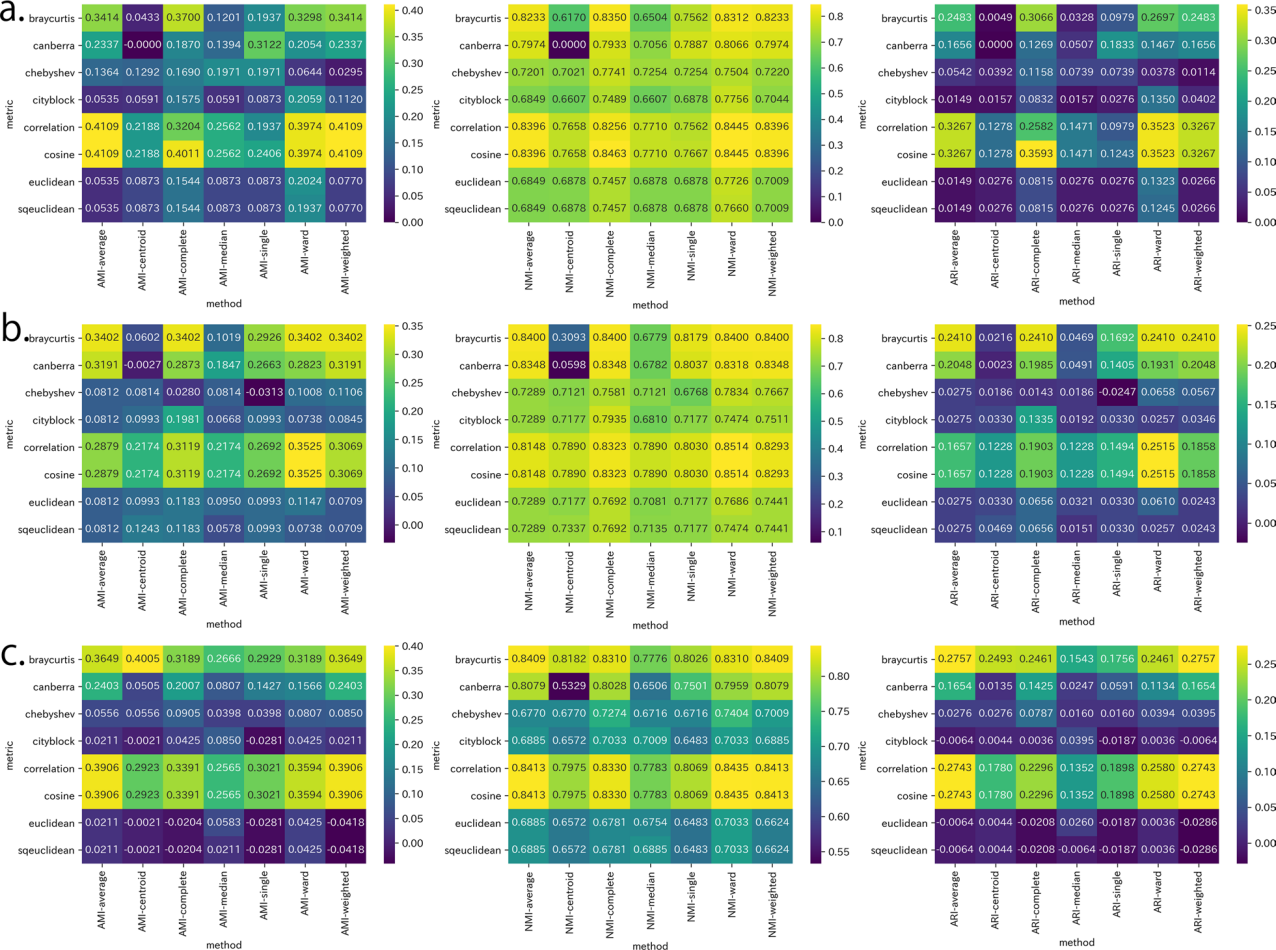
External validity scales for ICD-10 for embedding vectors from **a** Word2Vec, **b** FastText, and **c** GloVe

The evaluation using the external validity scale is shown in Fig. 2. For Word2Vec, the AMI was maximal at 0.4109 with the cosine or correlation metric and the average or weighted method, and the NMI and ARI were maximal at 0.8463 and 0.3593, respectively, with the cosine metric and the complete method. For FastText, the AMI, NMI, and ARI were maximal at 0.3525, 0.8514, 0.2515, respectively, with the cosine or correlation metric and when the ward method was used. For GloVe, the AMI was maximal at 0.4005 with the braycurtis metric and the centroid method, the NMI was maximal at 0.8435 with the cosine or correlation metric and when the ward method was used, and the ARI was maximal at 0.2757 with the braycurtis metric and the average or weighted method. Although the maximum metric and method differed, the generally high values for the cosine, correlation, and braycurtis metrics were similar for all the learning algorithms. Note that combinations with high ratings on the internal validity scale did not necessarily have high ratings on the external validity scale. Table 3 summarizes the metrics and methods that resulted in larger indicators.

**Table 3:** Summary of the metrics and methods that make each indicator larger with each algorithm

		Metric				Method			
		Highest average value				Highest average value			
		Maximize	1st	2nd	3rd	Maximize	1st	2nd	3rd
CCC	Word2Vec	Euclidean	Euclidean	Sqeuclidean	Cityblock	Centroid	Average	Weighted	Single
	FastText	Euclidean	Euclidean	Cityblock	Sqeuclidean	Average	Average	Weighted	Single
	Glove	Cityblock	Cityblock	Sqeuclidean	Euclidean	Average	Average	Weighted	Centroid
AMI	Word2Vec	Correlation/cosine	Cosine	Correlation	Braycurtis	Average/weighted	Ward	Complete	Weighted
	FastText	Correlation/cosine	Correlation/cosine		Braycurtis	Ward	Complete	Ward	Weighted
	Glove	Braycurtis	Correlation/cosine		Braycurtis	Centroid	Average	Weighted	Ward
NMI	Word2Vec	Cosine	Cosine	Correlation	Braycurtis	Complete	Ward	Complete	Weighted
	FastText	Correlation/cosine	Cosine	Correlation	Braycurtis	Ward	Complete	Ward	Weighted
	Glove	Correlation/cosine	Braycurtis	Correlation/cosine		Ward	Ward	Complete	Average
ARI	Word2Vec	Cosine	Cosine	Correlation	Braycurtis	Complete	Ward	Complete	Weighted
	FastText	correlation/cosine	Braycurtis	Correlation/cosine		Ward	Ward	Complete	Weighted
	Glove	Braycurtis	Braycurtis	Correlation/cosine		Average/weighted	Average	Weighted	Ward

**Fig. 3:**
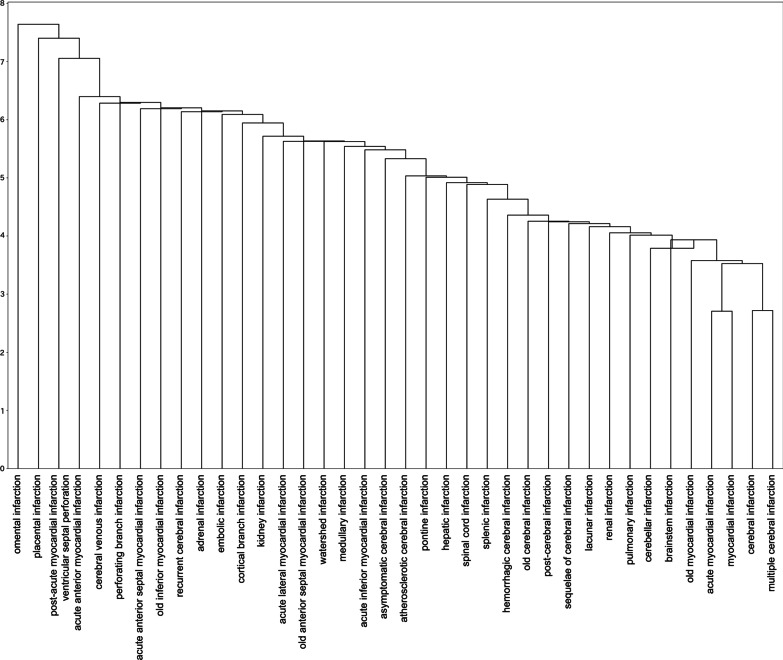
Dendrogram with euclidean and centroid that maximizes the internal validity measure. The vertical axis represents distance

**Fig. 4:**
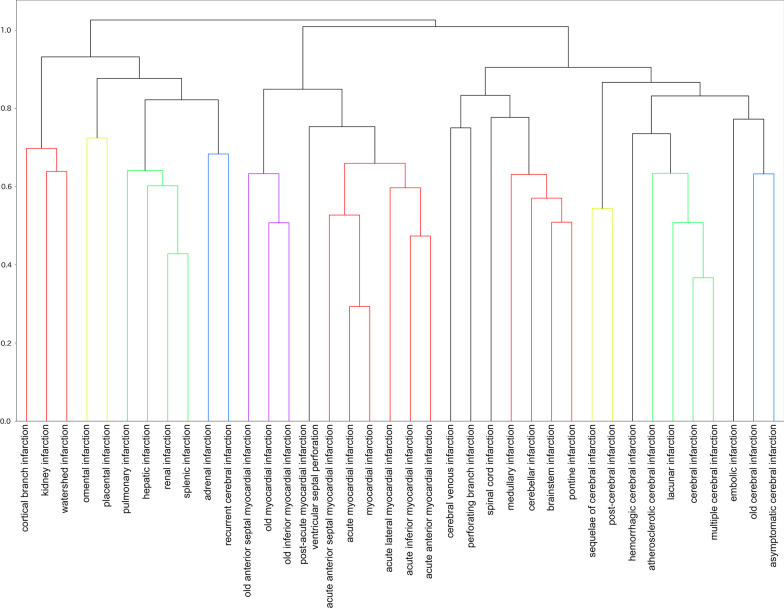
Six colored dendrogram when cosine and complete maximize the NMI and ARI. The vertical axis denotes distance, with 0.73 as the threshold

The dendrograms in Figs. 3 and 4, discussed in detail below, illustrate the combination of metrics and methods that performed best on the internal and validity scale evaluations for Word2Vec. In Fig. 4, we arbitrarily set thresholds and colors to make the figure easier to view.

## Discussion

We extracted abstracts related to “infarction” from a database of Japanese medical documents and used them as a corpus to obtain word variance representations using Word2Vec, FastText, and GloVe. The variance representation thus obtained allowed us to measure inter-disease distances, which indicate the degrees of similarity among diseases. Our examination of multiple metrics and methods revealed that the combination of the euclidean metric and the centroid method was optimal for assessing internal validity, while the combination of cosine distance and the complete linkage method was optimal for assessing external validity with ICD-10 for NMI and AMI when using Word2Vec. The inter-disease distances between word embedding vectors are, therefore, expected to be a valid quantitative representation of similar disease groups.

Word2Vec, FastText, and GloVe use deep learning based on the co-occurrence of words within a context to obtain a word embedding vector. Thus, words that appear in similar contexts have high similarity. In academic abstracts, the description of infraction in an organ also includes clinical symptoms and characteristic information derived from that organ. Differences in the characteristic information co-occurring in different organs may be a factor in the distance between diseases.

In the medical field, there have been few challenges to classification tasks with embedded representations. In a study that used a distributed representation obtained from medical records as visit embedding, the k-means method was used to classify the characteristics of patients by specialty [31]. A German Word2Vec model trained on a corpus of 352 MB of medical reports attained an accuracy of 90% in assigning medical reports written by physicians to ICD-10 [32]. The study identified rare diseases, unusual designations, and ICD code degeneracy as sources of assignment or “missing” errors. ICD-10 has a hierarchical structure with more than 68,000 codes. However, not all codes have the same level of granularity. Furthermore, ICD codes that are ontologically distant are less likely to be grouped [29]. Although we have bound our embedding vectors to ICD-10 based on the corpus of academic literature, the maximum value of NMI is 0.85 but only 0.41 and 0.36 for AMI and ARI when using Word2Vec; therefore, our embedding vectors cannot be interpreted as a mapping to the continuous space of ICD-10.

Note that the metric and method that maximized the internal validity measure and those that maximized the external validity measure produced different results. The euclidean maximized the internal validity measure but did not maximize the external validity rating. The dendrogram with the parameter that maximizes internal validity (Fig. 3) gives the impression that, unlike the ICD-10 classification, the less clinically relevant diseases are adjacent. Conversely, the dendrogram with parameters that maximize the NMI and ARI with ICD-10 (Fig. 4) gives the impression of a classification based on anatomical and temporal differences such as “old” and “sequelae.” In other words, the latter could be classified as “ICD-10-like.”

The Euclidean distance is calculated between two points as follows:

2 $$ d_{ij} = \left[ {\sum\nolimits_{k = 1}^{K} {\left( {x_{ik} - x_{jk} } \right)^{2} } } \right]^{1/2} ,\; {\mathbf{x}}_{i} = \left[ {x_{i1} , x_{i2} , \ldots ,x_{iK} } \right]^{T} $$

$$ d_{ij} :distance between {\textbf{x}}_{i} and {\textbf{x}}_{j} ,{\textbf{x}} \in {\mathbb{R}}^{K} , $$

$$ x_{iK} :k - th component of {\mathbf{x}}_{i} , {\mathbf{x}}_{i} : i - th component of {\mathbf{x}} $$

Conversely, the cosine distance is calculated from the angles between the vectors as

3 $$ d_{ij} = 1 - \frac{{{\mathbf{x}}_{i} \cdot {\mathbf{x}}_{j} }}{{{\mathbf{x}}_{i} {\mathbf{x}}_{j} }}, {\mathbf{x}}_{i} = \left[ {x_{i1} , x_{i2} , \ldots ,x_{iK} } \right]^{T} $$

When performing calculations with cosine distance, the length information of the vectors is lost because it is divided by the L2 norm. In other words, when the angle between two vectors *θ* is 0°, the cosine distance is 0 (similarity is 1) even if the L2 norm is different; therefore, diseases at different distances from the origin (L2 norm) in a higher-dimensional space are expressed as being similar. The L2 norm of **x**_*i*_ is calculated using the formula

4 $$ L_{i}^{2} = \left\| {{\mathbf{x}}_{i} } \right\| = \left[ {\sum\nolimits_{k = 1}^{K} {\left( {x_{ik} } \right)^{2} } } \right]^{1/2} $$

$$ L_{i}^{2} :L2 norm\;of\;the\; i - th\;component\;of\;{\mathbf{x}} $$

When clustering by cosine distance, all disease embedding vectors are normalized and plotted on the n-dimensional hypersphere, losing the L2 norm. Pivot and cluster were converted into a two-dimensional representation and shared with others, but this could also be represented on the hypersphere. A cluster is a group of diseases that make an angle of *θ* < *θ*_0_ with the pivot, and the inter-disease distance can be defined as the distance between two points on the hypersphere.

**Fig. 5:**
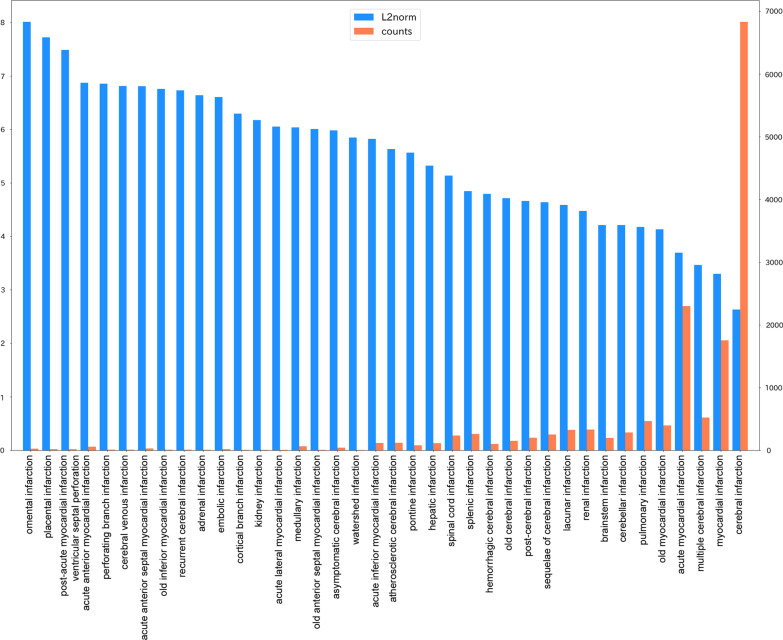
L2 norm and word counts of embedding vectors

Conversely, the L2 norm is not negligible. Vectors represent words that are consistently used in similar contexts with larger L2 norms than words of the same frequency used in different contexts [33]. Applied to diseases and symptoms, medical words that denote various causes are highly abstract and are used in various contexts (e.g., “infarction” and “headache”) have a smaller L2 norm. By contrast, medical words that are less diverse in terms of the cause, are more specific, and are used in limited contexts (e.g., “omental infarction” and “placental infarction”) have a higher L2 norm. In fact, the L2 norms of the embedded vectors in this study are “infarction”: 3.107, “headache”: 3.955, “omental infarction”: 7.703, and “placental infarction”: 7.583. The L2 norm of the standard disease list is shown in Fig. 5. The frequency of occurrence and the L2 norm tend to be inversely proportional.

The similarity with the dendrogram shown in Fig. 3 is also clear. Because the L2 norm is a Euclidean distance, this dendrogram reflects the frequency of occurrence and context of words in the corpus. Thus, using an unnormalized vector for classification may be preferable for considering word frequency and polysemy and to apply PCS in actual clinical practice.

The word frequencies should be limited to and interpreted within the corpus used in this study. However, using a broad corpus of academic medical literature may, in principle, be consistent with disease frequencies in the real world. Prior probabilities are essential information in clinical reasoning. By using Bayes’ theorem to modify the posterior probability of a diagnosis when new information becomes available, the prior probability represents its starting probability. In many cases, the prior probability depends on the function and location of the medical facility. By leaving the L2 norm to obtain clusters, a differential diagnoses list that considers prior probabilities in the corpus or facility may be obtained. By using different clusters for different stages of clinical reasoning (varying the distance and updating method), such computation may provide a more efficient differential diagnoses list that is more in line with the physician’s thought process.

Although this study was limited to the infarction domain, depending on the dictionary used in the morphological analysis, we simultaneously obtained the embedded vectors of medical words other than infarction disease. In other words, we efficiently computed vectors that represent symptoms, such as hemiplegia and headache, and histories such as smoking and hypertension. In the future, we will examine validity scales for domains other than infarction and calculate inter-symptom distances or symptom-disease distances to visualize many keywords used in clinical reasoning.

Bidirectional encoder representations from transformers (BERT) is a pre-training transformer-based machine learning technique developed by Google [34]. BERT has exhibited good performance on several natural language understanding tasks. The corpus used in this study was too small to create a pre-training BERT model and was not in a form suitable for fine tuning. Consequently, we could not use BERT. The use of BERT will be considered in future studies using large corpora.

### Limitations

There are several limitations to this study. First, we cannot assert that the corpus size used for the study was sufficiently large. Previous studies using academic literature corpora have acquired over 18 million abstracts to obtain a vocabulary of approximately 7.8 million words [35]. Ichushi Web is the most extensive Japanese-language academic corpus currently available, and this study used all the searchable medical journals available therein. Therefore, other resources should be considered to increase the corpus size.

Second, morphological analysis presents a problem. Many medical terms consist of multiple words, which is also true in Japanese; for example, “acute inferior myocardial infarction” contains four words in English and eight Chinese characters but is a single medical term in both languages. Word2Vec and GloVe are vulnerable when they encounter unknown words, and if a term is not entered as a multi-word term in the dictionary, it is divided similarly to the longest words. For example, if the medical term “acute right renal infarction” is present in the corpus but not in the dictionary, it will be divided into “acute,” “right,” and “renal infarction.” For morphological analysis, this study used the ComeJisyo medical dictionary, which, since November 2018, has 75,861 registered words. We can use the pre-trained model in Japanese with Word2Vec and FastText, but not with GloVe. However, some pre-training models for Japanese do not specify a dictionary for word segmentation. Nevertheless, depending on the domain and task, the dictionary registration of multi-word terms is inadequate, as found in this study. For the PCS task in the medical domain, definitively stating whether using a pre-trained model with a general domain corpus or a model learned with the medical domain is better is beyond the scope of this study.

Third, these technologies present a problem. Because they use random numbers during training, minute differences may occur each time the training is conducted, and the reproducibility of the study cannot be adequately guaranteed. In addition, because we did not mention the differences in research results that are due to differences in parameters, it cannot be asserted that the parameter settings used in this study are optimal.

Fourth, the ICD-10 classification prepared as an external validity measure is sometimes inappropriate for creating a clinical differential diagnosis list/cluster. Conversely, the true pre-prepared clusters mentioned in the previous study are not always found in textbooks or international classifications. If a list of physicians’ definitive differential diagnoses exists, it may be worth examining external validity scales on that list.

## Conclusions

The word embedded vectors produced by Word2Vec, FastText, and GloVe, trained on the infarction domain from a Japanese academic medical corpus, allowed us to represent the objective similarity between diseases (inter-disease distance) that can be used in PCS. Internal validity tended to be maximized when the metric was euclidean. There was no commonality among the three algorithms regarding the metric and method that could maximize AMI, NMI, and ARI. When using Word2Vec, the indices as the external validity scale with ICD-10 tended to be maximized when the metric was cosine. When the frequency of word occurrence is considered, the representation of the inter-disease distance by Euclidean distance, which does not ignore the L2 norm of the embedding vector, may be a quantitative index in implementing PCS. When clinical differences similar to ICD-10 are considered, the inter-disease distance expression may be that of using the cosine distance.

## Data Availability

The data used in the analysis can be downloaded from Ichushi Web, but the Japan Medical Abstracts Society (Ichushi) owns the rights to the data and cannot provide them through the authors. Embedded vectors of the training results may be provided by the authors if the Japan Medical Abstracts Society permits. The scripts used in the analysis can be provided by the corresponding author upon reasonable request.

## Abbreviations

AMI: Adjusted mutual information
ARI: Adjusted rand index
BERT: Bidirectional encoder representations from transformers
CBOW: Continuous bag of words
CCC: Cophenetic correlation coefficient
ICD: International statistical classification of diseases and related health problems
NMI: Normalized mutual information
PCS: Pivot and cluster strategy
